# An undergraduate laboratory experiment with real‐world applications: Utilizing templateless polymerase chain reaction and real‐time polymerase chain reaction to test for SARS‐CoV‐2 RNA

**DOI:** 10.1002/bmb.21593

**Published:** 2021-12-04

**Authors:** Julia Crane, Shallee T. Page

**Affiliations:** ^1^ College of Health and Natural Sciences Franklin Pierce University Rindge New Hampshire USA

**Keywords:** laboratory exercise, RT‐PCR, SARS‐CoV2

## Abstract

We present a course‐embedded undergraduate research module that involves real‐time polymerase chain reaction testing for the presence of SARS‐CoV‐2 in environmental samples. A positive control RNA was constructed and two RNA extraction methods were compared and a range of primers were available to compare. Using a combination of published protocols, we assembled a successful project that illustrated a topical exercise similar to real‐world assay development. The exercise is aimed at upper‐level undergraduates and requires 3 weeks of laboratory periods. The students were able to design and test experimental protocols, while learning about RNA detection. This project could be utilized in upper‐level classes including molecular biology, biochemistry, biotechnology, or for independent research projects.

## Graphical abstract

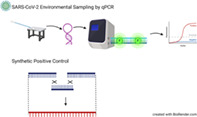

## 1 INTRODUCTION

Since March 11, 2020, when the World Health Organization declared COVID‐19 a pandemic, the entire globe has been scrambling for answers about the virus SARS‐CoV‐2 (short for severe acute respiratory syndrome coronavirus 2). As of October 11, 2020, over 36 million cases of COVID‐19 and 1 million deaths have been reported.^1^ Much of the scientific research community's efforts have been directed toward finding the cheapest, fastest, and most effective way to detect a positive case of COVID‐19.^2^, ^3^ Effective testing allows for contact tracing and appropriate quarantining measures, which could curtail the spread of the virus.^4^ Undergraduate biochemistry students at Franklin Pierce University sought to perform their own COVID‐19 tests on environmental samples from the Rindge campus. This three‐week laboratory module provides instruction in molecular tools such as real‐time polymerase chain reaction (RT‐PCR) as well as the process by which a clinical diagnostic test can be designed and executed.

This laboratory experiment follows the format of a course‐based research experiences (CURE). Courses in biochemistry and molecular biology are enriched by course‐based research experiences. The U.S. federal “Engage to Excel” report recommended widespread adoption of active learning through problem‐based learning such as CURE laboratories^5^ (Gates, 2012). CUREs have been shown to increase learning,^6^ increase persistence in science majors^7^, ^8^ as well as having positive impacts on appreciation for science and career aspirations.^9^ Some research even supports the benefits of CUREs when the research goals are not met.^10^

RT‐qPCR is a technique whereby RNA is converted to complementary DNA (cDNA) by reverse transcriptase enzyme, followed by quantitative PCR (qPCR). As with standard PCR, qPCR involves amplification of DNA using cycles of denaturation, annealing, and elongation. However, with qPCR, the presence of a fluorescent dye that binds double‐stranded DNA (dsDNA) allows for quantitation. The greater the amount of RNA, the more cDNA created and the fewer doubling cycles before the fluorescence can be detected.^11^ Thus, a smaller number of cycles to detect fluorescence, the more RNA in the original sample. A number of articles have been published that provide undergraduate exercises using qPCR or RT‐PCR. While some of these sources use the acronym RT‐PCR to stand for real‐time PCR,^12^, ^13^ others use RT‐PCR to stand for reverse transcription PCR^14^ and some just refer to the process as qPCR.^15^ Some COVID testing protocols use the term RT‐qPCR,^16^ but our procedure is not a truly quantitative PCR (qPCR) because we did not calculate starting RNA calculations nor use a housekeeping gene for comparison of gene expression; thus, we will refer to the technique as RT‐PCR. In our experiment, students investigated the presence of SARS‐CoV‐2 RNA on high‐touch surfaces by swabbing them and performing RT‐PCR on the samples.

RT‐PCR has been the most common diagnostic measure employed to detect the presence of SARS‐CoV‐2 on nasopharyngeal swabs.^17^, ^18^ To use this method as a diagnostic tool for SARS‐CoV‐2 RNA, a fluorescence threshold (C_T_) is defined. If the sample reaches the fluorescence threshold in a small number of PCR cycles (low C_T_), that sample is positive for SARS‐CoV‐2 RNA, as it indicates that there was a larger amount of RNA in the original sample. If C_T_ is very large, or if it never reaches the fluorescence threshold, that sample is negative for SARS‐CoV‐2 RNA. Correlations between disease progression and C_T_ values have been established,^19^ as well as correlations with culturable virus.^20^, ^21^

This paper describes the process of how the undergraduate biochemistry class designed and ran their own RT‐PCR tests to detect the possible presence of SARS‐CoV‐2 on campus. This lab was designed to demonstrate RT‐PCR to the undergraduate biochemistry class, as well as to potentially detect samples that were positive for SARS‐CoV‐2 on campus. The synthetic positive control was based on a procedure by Meza‐Robles et al for one‐step nested SARS‐CoV‐2 RT‐PCR.^22^ This exercise utilizes no virus, so it involves no additional safety concerns. Pedagogical advantages of the experiment include problem‐based learning, experimental design, clinical applications and a real‐world application.

## 2 PREREQUISITE KNOWLEDGE AND LEARNING OBJECTIVES

Students participating in this exercise should have passed undergraduate general biology. They should have a basic understanding of the central dogma of biology, transcriptional machinery and the process of polymerase chain reaction. The students need not have prior knowledge or experience with RT‐PCR. Instructors should have some experience with basic molecular techniques and RT‐PCR and access to instrumentation.

ASBMB lists a number of desirable learning goals associated with understanding and manipulation of DNA and RNA, as well as experimental design and data analysis. These learning goals are foundational concepts under the category of “Information Storage and Transfer.”^23^ Our observable learning objectives for this research module were that the student will be able to demonstrate understanding of the molecular biology, plan and design an experiment based on primary literature, develop a procedure that meets assigned goals, execute their experimental plan in a collaborative fashion, and analyze and communicate the results.

## 3 TIMING

This lab was administered in a sequential fashion where each lab group collected samples, but they collaborated vertically on the procedure and no groups completed all steps. This was partially necessitated by the need to socially distance in the laboratory sections due to the COVID pandemic. As a result, we had three to four lab sessions a week and the project lasted less than 4 weeks. The timetable below reflects the number of 2‐hour sessions required to complete the lab as though one group had completed them all sequentially. Other timing configurations are also possible: an instructor could have one group create the positive control at the same time that another group ran the reverse transcription, or have a lab assistant prepare the gel in advance. Depending on class size, group size could range from two to four students.

Four weeks before the lab began, we obtained the price quote for the primers and began ordering. The three oligos for the positive control were the most expensive (~$115 each). The week before the lab began, informational materials were posted (Supporting Information). Note that this requires students to spend a week reading over the materials and plan out their experiments. To orient the groups, prelab lectures always included a review of the flow chart of the entire project (Supporting Information). In reality, a minority of students had carefully read the materials and prepared a full experimental protocol. However, they were able to ask questions and clarify their understanding during lab period one since it was just buffer preparation and sampling.

*Lab 1*—During lab period one, the students prepared buffers, labeled tubes, distributed sterile swabs and each student swabbed a campus location. Lab 2—Students prepared the templateless PCR to create the positive control. *Lab 3*—Students took the thawed samples and ran the reverse transcription reaction. *Lab 4*—Students poured the agarose gel and made the buffers and prepared the electrophoresis gel. *Lab 5*—Students loaded the gel with the ladder and positive control, ran the gel, stained it with SYBRSafe and visualized it. *Lab 6*—Students set up the RT‐PCR, and ran the instrument.

The students were given the expectation that their procedure and any findings were posted to the shared electronic lab notebook within 24 h so that other groups could utilize that information to proceed. This did not always occur; however, the lab report included a list of the distribution of work, and a peer evaluation protocol to help the instructor recognize students' contributions.

## 4 PROCEDURE

### 4.1 Materials

Materials needed for the research module included synthesized oligonucleotides and primers, typical molecular biology reagents and equipment and RT‐qPCR instrumentation. Students were not given a lab procedure. Instead, for an inquiry lab, they were provided with background information and primary literature (Supporting Information). A sample student protocol is provided (Supporting Information).

### 4.2 Sampling

The CDC Diagnostic Panel provides extensive guidance on sampling, handling and preserving samples, primer choice, reagents used, and procedure.^24^ We only followed their procedures loosely. Students could choose a physiological buffer with tetracycline antibiotic. Swabs were dipped in buffer, wiped twice over a surface, then broken off into the collection tube and capped. Collected samples were stored at −80°C until use.

### 4.3 Generation of the positive control

**FIGURE 1:**
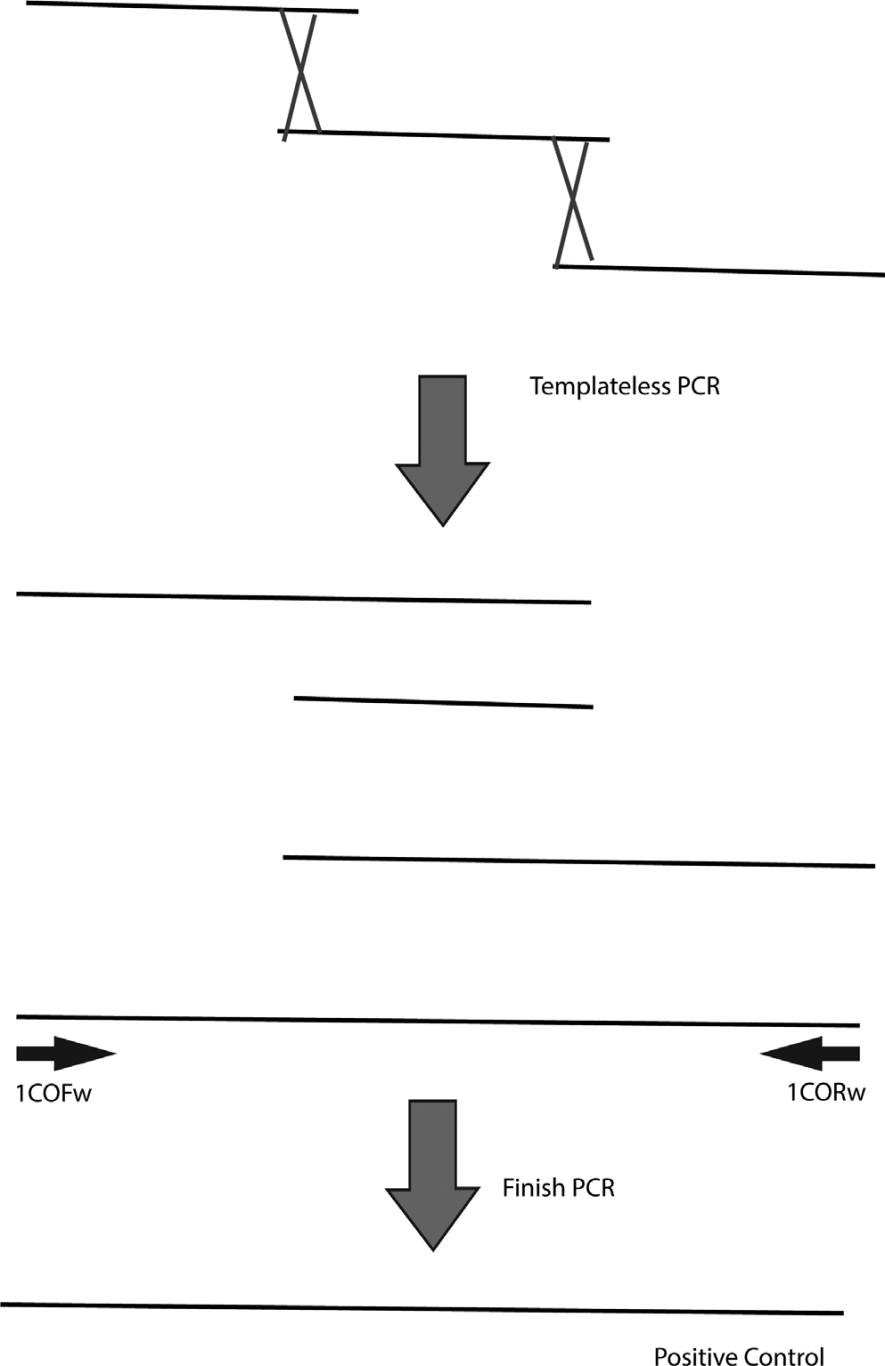
Cartoon illustrating templateless polymerase chain reaction (PCR). Three oligos were synthesized (IDT) according to Meza‐Robles et al.^22^ with overlapping sequences. When joined by Taq polymerase, they assemble into a contiguous strand in low efficiency, resulting in a mixed population of products. Finish PCR is then performed to select for the desired product using primers (1COFw and 1CORw) complementary to the ends of the complete product. This amplifies the positive control, in this case a partial sequence of SARS‐CoV2 ORF1a

A synthetic, non‐infectious sequence was generated to serve as a positive control, based on a paper by Meza‐Robles et al.,^22^ using templateless PCR to create a large positive control sequence based on the SARS‐CoV2 ORF1ab gene sequence. Three oligonucleotides (1COV, 2COV, 3COV) were synthesized (Table S1) and assembled by templateless PCR. Templateless PCR relies on overlapping oligonucleotides in a PCR reaction with polymerase master mix to generate a contiguous sequence (Figure 1) that can be then detected by PCR. The sequence and cycling conditions were as utilized in Meza‐Robles^22^ and then, the product was re‐amplified using nested primers under the same cycling parameters. The resulting fragment served as a synthetic positive control for the ORF1ab gene of SARS‐CoV2 and was stored at −20°C. The original product of the 1COV‐2COV‐3COV templateless PCR can also be used as a positive control with the 1COFw and 1CORw primers, but the yield of the templateless PCR is typically low. Note that Meza‐Robles^22^ constructed their positive control in a one‐step nested PCR, but we performed it in two steps, with no intermediate purification, for the sake of simplicity.

### 4.4 Gel electrophoresis

Electrophoresis of the positive control was run on a 2.5% agarose gel with the positive control generated by templateless PCR, negative control, and a ladder. The positive control PCR product was purified from the gel slices using a spin‐column gel extraction kit according to manufacturer's instructions.

### 4.5 RNA extraction

The intention was to have the students compare our RNA purification kit to heat extraction to release the RNA from the virus. The students were provided with technical information for the RNA purification kit and a journal article that reported the success of the heat release in the absence of RNA purification.^16^ However, the first group of students tried the heat release and it worked well, so they all chose heat extraction.

### 4.6 First‐Strand synthesis and RT‐PCR

Using a purchased kit, first‐strand synthesis (FSS) was completed to generate cDNA that could then be amplified in RT‐PCR. Each environmental sample was reverse transcribed in addition to one negative control per group that contained no enzyme. DNA generated from FSS and the positive control were run through RT‐PCR. The thermocycler was set to run for 40 cycles with an annealing temperature of 53°C. A melt curve was conducted with SYBR‐Green fluorescence data collection after each 0.5°C increase in temperature.

### 4.7 Safety and waste disposal

Although the samples used were environmental samples—and even though some materials, such as the positive control and electrophoresis waste, are not hazardous—all products from the experiment were collected as biohazard and autoclaved in order to eliminate the possibility of COVID‐positive environmental samples being disposed of inappropriately. Goggles, gloves, and cloth or surgical masks were worn for the duration of the experiment. A HEPA air filter ran continuously and hand sanitizer was available.

## 5 RESULTS

The laboratory successfully detected SARS‐CoV‐2 RNA, as judged by C_T_ values (Table 1). Both laboratory sections detected high concentrations of the positive control (C_T_ 1–2 cycles). Both sections had environmental samples that tested positive for SARS‐CoV‐2 RNA, mostly low levels that were unlikely to be infectious. One environmental sample did have a C_T_ value of 23, which could be consistent with an infectious viral load.^19^

**TABLE 1:** RT‐PCR results

Well	Fluorophore	Sample	C_T_	Melt temperature (°C)
F01	SYBR	None	NaN	None
F02	SYBR	None	NaN	None
F03	SYBR	+	1.008959	74.5
F04	SYBR	None	NaN	None
F05	SYBR	Sample 3	23.10921	75.5
F06	SYBR	−	NaN	None
F07	SYBR	−	NaN	None
F08	SYBR	None	NaN	None
F09	SYBR	Sample 2	31.38888	74
F10	SYBR	Sample 1	31.79547	75.5

*Note*: RT‐PCR Data. Data shown is from the section with the most positive samples. Well indicates arbitrary sample position; Fluorophore SYBR is SybrGreen; Sample‐(+) indicates synthetic positive control; Sample‐(−) indicates—reverse transcriptase negative control or—template negative control. C_T_ indicates the number of cycles to reach threshold detection; Melt temperature is the temperature for the dsDNA to denature.

Abbreviation: RT‐PCR, real‐time polymerase chain reaction.

The melt curves showed a drop in fluorescence at similar temperatures, as the double‐stranded PCR product strands separated (Figure S1A). Those same data are also shown as a change in slope of the curve, resulting in a peak at the “melt temperature” (Figure S1B). Because the melt temperature depends on the guanine‐cytosine content (and distribution) of the product, overlapping peaks are consistent with identical PCR products.

## 6 CHALLENGES

We have only run this lab exercise once, but we were able to successfully complete the module with few difficulties. With many molecular biology lab exercises, the most common difficulties arise from poor use of micropipettors or use of incorrect reagents. This is particularly important for PCR. Most of our biochemistry students have had two previous courses using micropipettors, but we still have better success when we require successful completion of a lab module calibrating the micropipettors at the beginning of the semester. Students with previous exposure to quantitative transfer of small volumes and PCR should be able to successfully complete the lab.

As was evident from the prelab quiz and lack of progress at the beginning of the period, some students did not prepare at all before the lab period which, unsurprisingly, inhibited their ability to process the procedures. Almost all of the students struggled to understand the role and mechanism of the templateless PCR. The difficulty was compounded by the fact that some groups did not participate in the hands‐on templateless PCR preparation. We recommend an individual tutorial (Figure 1) or explanatory video on that technique alone (e.g., Reference 25) or, when possible, have at least one group in each section perform the templateless PCR.

The most striking difficulty students encountered, though, is a ubiquitous one with undergraduate group projects: some of the students did not contribute equally to the communication, writing, and revision process. Two groups had to be reminded to post their data. Because students were posting in their electronic notebooks individually, individual grades reflected their effort (approximately 5% penalty for the lab, where notebooks were worth 30% of their course grade), but it created frustration for lab partners.

## 7 STUDENT WRITE‐UP

Before the first lab, students completed a prelab quiz. Our students conducted a single RT‐PCR run per lab section, so they were required to write up a single lab report per section. Our assessment utilized grading of individual lab notebooks (just the flow chart and the section the student pair worked on) and a joint lab report for the lab section. The joint lab notebook followed the format of a typical brief, formal lab report (Abstract, Introduction, Methods, Results, Analysis, Conclusions).

## 8 STUDENT SURVEY FEEDBACK

Students participated in this and one other research module in the class. An abbreviated version of the CURE survey^9^, ^26^ was administered before (*N* = 12) and after (*N* = 20) the students completed the research modules. Between the pre‐course survey and the post‐course survey, there were only a few variables that shifted. The students apparently had a good sense in the pre‐survey that real science can proceed in a non‐linear fashion but few had participated in a course where the students had input into the experiment design (“little” or “no” experience 83%).

Following the research modules, student feedback was positive but not uniformly so. 80% of the students (*N* = 20) disliked doing the lab sequentially or writing a single lab report as a group. 85% of the students said that the projects did not affect their attitudes or plans about graduate study but 10% said that they were somewhat more likely to consider graduate education after doing the research experience and one (5%) said that it made them less likely to consider postgraduate research. 55% said it gave them a better sense of how scientists think about experiments and plan them out, while 45% said that the aspect that they learned the most from was analyzing original data.

Asked in an open‐ended question what they most enjoyed in the research experience, one student noted that they greatly preferred the basic labs on spectrophotometry to the research experience because they felt more confident in their understanding and ability to complete the work. However, common responses were that they liked the flexibility in scheduling (20%), learning new techniques (20%), learning how people are doing COVID testing “in the real world” (10%). One student noted that they felt that they were “…able to experience true scientific discovery in a professional lab setting.”

## 9 EXTENSION ACTIVITIES

A range of possibilities exist for extension activities. We discussed experimental design and how COVID testing was being conducted with our classes. But, a full exploration of sensitivity, specificity, sampling techniques and clinical variation could be incorporated.

Students could rigorously compare the heat extraction versus traditional RNA extraction using chloroform/isopropanol or a spin column kit. Using a single label (SYBR green) means that only one target sequence can be assayed at once because the fluorescent dye will bind to any dsDNA that is amplified; use of more than one fluorophore would enable multiplex RT‐PCR for more than one target in one tube.^11^ Alternatively, probes can be designed that have their own sequence specificity, decreasing the chance of non‐specific binding, particularly for eukaryotic transcriptomes.^27^

Positive controls could be generated for other genes (Supporting Information). The qPCR can be done quantitatively, as demonstrated by Hancock et al.^13^ or McCauslin et al.^15^ This would require better quantitation of the positive control and could include a spike‐in of a small amount of the positive control in sample duplicates to rule out interfering substances and verify quantitation. In future years, SARS‐CoV‐2 will be less of a health concern, but these methods could be easily adapted to coronaviruses such as HCoV‐OC43 that cause the common cold,^28^ or other mild viral pathogens by simply changing the sequences for the positive control and for the primers.

## 10 CONCLUSIONS

This research module provides upper‐level undergraduates the opportunity to design and safely conduct an experiment that combines molecular biology design with a real‐world public health application. Experimental procedures that have been previously encountered such as agarose gel electrophoresis are reinforced. The exercise also requires the students to compare published experimental procedures and incorporate them into their own design.

The ability to work with DNA and RNA is fundamental for all biologists and biochemists. The use of RT‐qPCR is a sensitive technique that is convenient for comparative gene expression. Although RNA‐seq is becoming more common and more affordable, RT‐qPCR is still widely utilized. RT‐PCR also has applications in assessing DNA yield and in forensic science.^12^

Templateless PCR, used to generate the positive control, is a technique whereby large oligos can be designed with overlapping terminal sequences and assembled into a contiguous single‐stranded product by PCR. The technique is important for synthetic biology and has been used with undergraduates to generate large partially synthetic chromosomal segments.^29^

The students had been warned that we might have no positive environmental samples and that any positive samples would only indicate RNA, not necessarily infectious virus, and not necessarily indicate that there had been enough viral load to be infectious. We did, however, have a sample that had high RNA levels consistent with infectious virus.^19^ Our findings were almost 2 weeks after sampling in areas that had regular antiviral cleaning, but staff ensured additional decontamination of the area. In addition, to avoid any rumors or concerns from students, administration, or parents, we also created a FAQ about our experiment and findings (Supporting Information).

The COVID‐19 test development module proved to be an effective teaching tool for modern molecular biology techniques. The topic was of extremely high interest for the students and their enthusiasm was palpable. Basic skills reinforced include viral physiology, transcription and reverse transcription, and experimental design. Topics that were novel to the students included viral sampling and storage, templateless PCR, and use of qPCR instrumentation. This RT‐PCR lab module provided a genuine research experience with current clinical applications of critical public health importance.

## Supporting information


**Figure S1** Melt Curves Following RT‐PCR
The qPCR cycler was programmed to conducting melting curve analysis following completion of the RT‐PCR. The cycler progressed from 55°C to 95°C with fluorescence measurements after each 0.5°C increment. Horizontal bars reflect negative controls and empty wells overlaid. (A) – Melt curve measuring fluorescence versus temperature. (B) – Identical data as above with the y‐axis indicating a change in slope of the curve.
**Figure S2** Flow chart of procedure.

**Table S1** Primers
