# Supplementary figures and images for: An undergraduate laboratory experiment with real‐world applications: Utilizing templateless polymerase chain reaction and real‐time polymerase chain reaction to test for SARS‐CoV‐2 RNA
Source: Biochem Mol Biol Educ. 2021 Dec 4;50(1):142–8. doi: 10.1002/bmb.21593 (PMC9011848; doi:10.1002/bmb.21593)

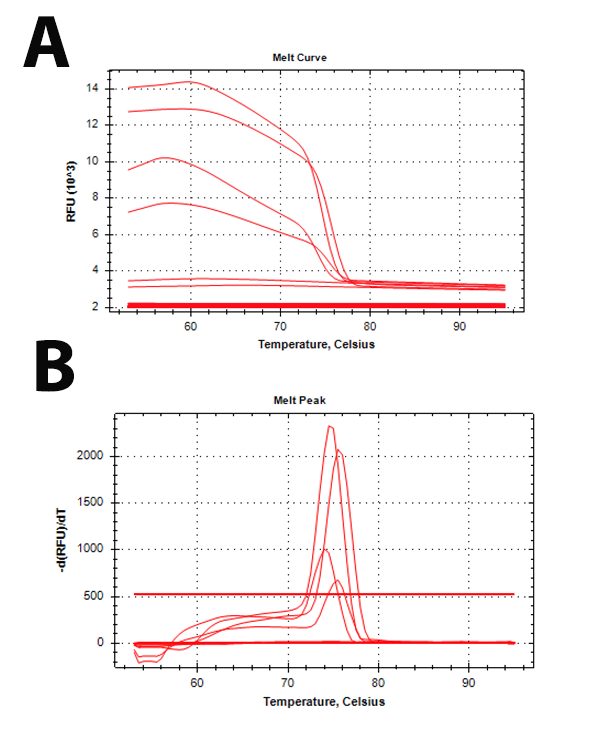

Supplement: Supplementary file 1 — Figure S1 Melt Curves Following RT‐PCR The qPCR cycler was programmed to conducting melting curve analysis following completion of the RT‐PCR. The cycler progressed from 55°C to 95°C with fluorescence measurements after each 0.5°C increment. Horizontal bars reflect negative controls and empty wells overlaid. (A) – Melt curve measuring fluorescence versus temperature. (B) – Identical data as above with the y‐axis indicating a change in slope of the curve. Figure S2 Flow chart of procedure. [file BMB-50-142-s002.tif]
