# Supplementary material for: An undergraduate laboratory experiment with real‐world applications: Utilizing templateless polymerase chain reaction and real‐time polymerase chain reaction to test for SARS‐CoV‐2 RNA
Source: Biochem Mol Biol Educ. 2021 Dec 4;50(1):142–8. doi: 10.1002/bmb.21593 (PMC9011848; doi:10.1002/bmb.21593)
Supplement: Supplementary file 2 — Table S1 Primers [file BMB-50-142-s001.docx]

SUPPLEMENTAL MATERIALS

1. Student resources
   1. Readings
   2. Flow chart
2. Instructor resources
   1. Purchasing
      1. Primers
      2. Materials
   2. Instructor Preparation
   3. Sample Student Protocol
   4. Administrator FAQs
3. Student resources
   1. Readings and resources

- A video from NOVA <https://www.pbs.org/wgbh/nova/video/how-coronavirus-tests-work/>
- A video introduction to SARS-CoV2 the lab concepts, diagnostic testing and general procedure, from the instructor available upon request. The prelab lecture was recorded via Zoom. The recording can be shared upon request.
- A qPCR handbook for reference. <https://go.idtdna.com/qpcr-designguide.html>
- Journal articles about COVID testing using RT-PCR.

Bustin et al, 2020 [1].

Park et al, 2020 [2].

Bruce et al, 2020 [3].

Kellner et al, 2020 [4].

Hasan et al, 2020 [5].

- We had four remote students who were put in charge of posting a brief summary of four of the journal articles. The fifth one was Meza-Robles, 2020 [6], our primary source for the positive control.
- Students were provided with information about CDC procedures for sampling and virus sample handling (<https://www.cdc.gov/coronavirus/2019-ncov/downloads/Viral-Transport-Medium.pdf> ).

Works Posted for Students

1 S. A. Bustin, T. Nolan RT-QPCR testing of SARS-COV-2: A primer. (2020) *Int. J. Mol. Sci.* **21**, 3004.

2 M. Park, J. Won, B. Y. Choi, C. J. Lee Optimization of primer sets and detection protocols for SARS-CoV-2 of coronavirus disease 2019 (COVID-19) using PCR and real-time PCR. (2020) *Exp. Mol. Med.* **52**, 963–977.

3 E. A. Bruce, M. L. Huang, G. A. Perchetti, S. Tighe, P. Laaguiby, J. J. Hoffman, et al. Direct RT-qPCR detection of SARS-CoV-2 RNA from patient nasopharyngeal swabs without an RNA extraction step. (2020) *PLoS Biol.* **18**,.

4 M. Kellner, J. Ross, J. Schnabl, M. Dekens, R. Heinen, I. Grishkovskaya, et al. A rapid, highly sensitive and open-access SARS-CoV-2 detection assay for laboratory and home testing. (2020) 1–28.

5 M. R. Hasan, F. Mirza, H. Al-Hail, S. Sundararaju, T. Xaba, M. Iqbal, et al. Detection of SARS-CoV-2 RNA by direct RT-qPCR on nasopharyngeal specimens without extraction of viral RNA. (2020) *PLoS One*. **15**, 1–9.

6 C. Meza-Robles, C. E. Barajas-Saucedo, D. Tiburcio-Jimenez, K. A. Mokay-Ramírez, V. Melnikov, I. P. Rodriguez-Sanchez, et al. One-step nested RT-PCR for COVID-19 detection: A flexible, locally developed test for SARS-CoV2 nucleic acid detection. (2020) *J. Infect. Dev. Ctries.* **14**, 679–684.

- 1. Protocols

Students were not provided with a protocol; they were expected to generate their own. A sample protocol is provided in Supplemental Materials under “Instructor Resources>Protocols”. The following flow chart was on the board for the duration of the project:

**Flow chart**

| Buffer preparation | |
| --- | --- |
| Sampling and storage  🡻 | Generation of Positive Control PCR  🡻 |
| RNA Extraction | Finish PCR  🡻 |
|  | Gel Extraction |
|  | |
| First-Strand synthesis (RT) | |
| Run RT-PCR | |
| Assess results | |

1. Instructor resources
   1. Purchasing
      1. Table S1. Primers

| **Local Name** | **Sequence** | **Target Gene** |
| --- | --- | --- |
|  |  |  |
| CoV2BruceN3F1 | GGG AGC CTT GAA TAC ACC AAA A | N |
| CoV2BruceN3R1 | TGT AGC ACG ATT GCA GCA TTG | N |
| COV2CDC-RPF1 | AGATTTGGACCTGCGAGCG | RP |
| COV2CDC-RPR1 | GAGCGGCTGTCTCCACAAGT | RP |
| China-1abF1 | CCCTGTGGGTTTTACACTTAA | 1ab |
| China-1abF1 | ACGATTGTGCATCAGCTGA | 1ab |
| Meza-1COFw | AGC AAG TTG AAC AAA AGA TCG CTG AG | 1ab |
| Meza-1CORw | CAA TAT AAA GTA ACA AGT TTT CTG TGA GG | 1ab |
| COV2CDC-N1F1 | GAC CCC AAA ATC AGC GAA AT | N |
| COV2CDC-N1R1 | TCT GGT TAC TGC CAG TTG AAT CTG | N |
|  |  |  |
| Positive Control |  |  |
| Meza-1COV | AGCAAGTTGAACAAAAGATCGCTGAGATTCCTAAAGAGGAAGTTAAGCCATTTATAACTGAAAGTAAACCTTCAGTTGAA | 1ab |
| Meza-2COV | AGTTTCTTCCAGAGTTGTTGTAACTTCTTCAACACAAGCTTTGATTTTCTTATCATCTTGTTTTCTCTGTTCAACTGAAG | 1ab |
| Meza-3COV | CAATATAAAGTAACAAGTTTTCTGTGAGGAACTTAGTTTCTTCCAGAGTTGTTGTAACTTCTTCAACACAAGC | 1ab |

N=nucleocapsid protein, RP=Ribonuclease P, 1ab=ORF1ab

- - 1. Materials – In addition to basic molecular biology supplies and thermal cyclers, the following were ordered:

|  | Company |  | Catalog # |
| --- | --- | --- | --- |
| RNA purification kit | Zymo | DirectZol | R2061 |
| qPCR master mix | BIORad | iTaq SYBR green supermix 200 RXN | 1725120 |
| Primers | IDT | Oligos 25 nmol |  |
| Large Oligos | IDT | Ultramer oligos 10 nmol |  |
| Ladder | New England Biolabs | Quick-Load® Purple 1 kb DNA Ladder | N0552S |
| Gel extraction | EZNA | spin-column gel extraction kit | D2500-00 |
| FSS kit | BioRad | iScript cDNA synthesis kit 25 RXNS | 1708840 |

Timetable

T-4 weeks –order primers and oligos

T-2 weeks - The lab assistant dissolved and aliquotted the primers. The primers were dissolved in 1 mL of sterile, nuclease-free distilled water and aliquotted to four tubes each.

Safeguards that should be utilized include careful labeling, color coding, sequestering reagents not in current use, and reserving aliquots that exceed that needed for the current experiment.

2-hour labs

Lab 1-Tubes, buffers, pH meters, acid & bases

Lab 2- Templateless PCR

Lab 3-RT

Before lab period three, samples were thawed and put on ice and the molecular biology reagents were aliquotted to avoid students inadvertently using up all of the reagents in one error. During lab period three,

Lab 4-Agarose equipment

Lab 5-Load and run gel

Instructor visualized the gel and circulated the image

Lab 6-RTPCR

Students were provided the output afterwards for analysis.

1. Sample Student Protocol

**Sample Student Protocol**

**(lightly edited by instructor for completeness and clarity)**

*Sampling (9/18) -Sample Collection:*

1.0 mL of a 15 mM Tris buffer with 1X tetracycline solution (final concentration 10 μg/mL) was made.

C_1_ V_1_ = C_2_ V_2_

(0.1M)(V1) = (15mM)50mL

(100mM)(V1)=(15mM)50 mL

V1=7.5 mL of Tris buffer

42.5 mL of molecular grade DI water

50 microliters of 1000X tetracycline (final concentration 10 µg/mL)

After buffer dilution was made but before the tetracycline was added, the pH was tested and was found to be too high. One drop of 5% HCl was added to bring down the pH to 7. The original buffer used was from Kelly's group. It had an original concentration of 0.1 M, and a pH of 7.45. Buffer was placed into 2 mL sterile microfuge tubes and kept on ice.

Cotton swabs that were sterilized in the autoclave were used to collect samples. The cotton swab was wetted with the buffer solution before sample collection. The wet cotton swab was wiped across the surface of interest. The CDC recommended swab surface area is 25 cm^2^. The tip of the swab was then broken off and kept in a tube containing the buffer solution that was used to wet that swab originally. This was repeated for each sample and samples were stored at –80 degrees Celsius until qPCR was done.

*Sample locations:*

[redacted]

These were labeled 1-12 and put into the –80 freezer to prevent RNA degradation.

*Positive Control (9/18):*

We centrifuged each oligo to get to bottom of tube.

We prepared a 100 µM stock solution for each oligo by adding 200 µL of phosphate

buffer to resuspend the pellets.

We diluted each solution 1:5 (20 µM) by adding 10 µL of the 100 µM stock solution to 40

µL phosphate buffer.

We prepared a 25 µL solution in a PCR tube by using 2.5 µL of BioRad SYBR Green 2X

Master Mix and 2 µL of each 20 µM oligo (1COV, 2COV, 3COV), 2 µL of taq

polymerase, 2.5 µL dNTPs and 12 µL DI water

We ran the thermal cycler for:

1 cycle at 94°C for 2 minutes

35 cycles at 94°C for 30 seconds

35 cycles at 53°C for 30 seconds

35 cycles at 72°C for 30 seconds

Samples were placed in –20°C freezer

*Finish PCR*

The positive control oligo was obtained from lab instructor and placed on ice. Inside two tubes, 10 μL of 2X Master Mix (buffer, enzyme, and dye), 1 μL of positive control from previous PCR, 2 μL of forward 1COVFw primer, 2 μL of reverse 1COVRw primer, and 5 μL of molecular grade H_2_O was placed. The concentration of the primers were very close to the necessary concentration so no dilution was needed. Both tubes placed in the thermal cycler, and run at 53°C for 5 minutes, and 94°C for 10 minutes. Then, the positive controls were run for 38 cycles at 94°C for 30 seconds, 53°C for 30 seconds, and 68°C for 30 seconds. Using the Nanodrop, we checked the RNA concentration and purity.

*Preparing Gel Electrophoresis: 9/22*

Adding Buffer:

• Using 50X TBE buffer, 5ml was added to a beaker, and 245ml of DI water was added to the buffer to make 50ml of 1X buffer

*Making a 2.5 % Agarose Gel:*

• We used 2.5g of agarose when I was only supposed to use 0.625g, added it to 25ml of DI water. We microwaved it and realized it was not the right amount of agarose so we added a lot more water to it. It was microwaved again until the substance was fully liquid. We then taped off the gel plate and poured the agarose mixture into the gel plate and let sit until solid. Since this was just a blank gel, the amount of agarose wasn't a problem.

Gel electrophoresis of the positive control was run on the 2.5% agarose gel in TAE buffer with the negative control, positive control generated by templateless PCR, and a ladder. For both the ladder and the positive control, 2 μl of sample, 2 μl of 6x dye, and 8 μl of dH_2_O were used; for the templateless PCR, 5 μl of sample, 2 μl of 6x dye, and 5 μl of dH2O were used. Samples were loaded into the gel and run at 50 V for 10 minutes, then 75 V for 50 minutes. SYBRSafe was added to the gel and results were visualized. The positive control was overloaded and ran as a large band smeared from 100-350 bp. The entire slice was cut out filling four Eppendorf tubes. The positive control PCR product was purified from the gel slices using a spin-column gel extraction kit (E.Z.N.A.# D2500-00) according to manufacturer’s instructions.

*RNA Isolation*

We heated to activate the environmental samples (previously collected) at 65°C for 10 minutes then room temperature for 10 minutes. Using the Nanodrop, we checked the RNA concentration and purity.

*First Strand Synthesis (9/25):*

After heat extraction, we created a solution for each 2.5 µL of each collected RNA sample from each group to the sterile nuclease-free microcentrifuge tubes after applying it to the vortex, then adding 5 µL 2X buffer, 1 µL 10X RT enzyme solution, 1.5 µL random primer mix to a centrifuge tube and mixed. One of the tubes had no RT enzyme.

Each tube was spun on a vortexer then placed in a 25°C water bath for 10 minutes

We placed the solutions in a heat bath at 42°C for 1 hour (plus an additional 40 minutes due to the heat bath not being turned on).

*RTPCR (10/2)*

We created six 0.5 mL PCR tubes each containing 3 µL sample, 1 µL of forward primer, 1 µL of forward primer, and 5 µL 2X Master Mix (buffer/dye/Mg/dNTPs/enzyme).

We created one negative control tubes by using 1 µL of the previously created negative control and 2 µL DI water, 1 µL primer 1, 1 µL primer 2, 5 µL 2X Master Mix. Created a second negative control by replacing the template and DI water with 3 µL of DI water.

We created a positive control tube using 1 µL sample. The PCR tube for the positive control was prepared with 2 μl nuclease free water and 1 μl of approximately 100 ng (as assayed by Nanodrop) positive control of the positive control and 2 µL DI water, 1 µL primer 1, 1 µL primer 2, and 5 µL Master Mix. All PCR tubes were vortexed and centrifuged, and placed in a BioRad CFX-96 thermocycler. The results were recorded and sent to us.

We placed all tubes in the qPCR machine and ran the program “COVID-MC”. The thermocycler was set to run at 95°C for 30 seconds, then run through 40 cycles of 10 seconds at 95°C followed by 30 sec at 53°C. A melt curve was conducted (55-95°C) with fluorescence data collection after each 0.5°C increase in temperature. The data were opened with Excel.

SAFETY

In addition to the splash-proof safety goggles, students wore personal protective equipment consistent with university pandemic precautions: lab coats, gloves, and personal face masks. Collection of environmental samples thus posed less safety hazard than typical casual movement about campus. Although the samples used were environmental samples, all products from the experiment were collected as biohazard and autoclaved.

1. **Administrative FAQs**

**Biochemistry SARS-CoV2 Project**

**What was the project?**

The students went through an authentic research experience that mimicked what public health officials had to wrestle with this spring: developing testing for SARS-CoV2.

**What were the students investigating?**

They were testing two different ways to extract RNA from environmental samples and several different primer sets (used in China, US CDC, Mexico).

**Are you doing COVID testing?**

No. We are testing for virus RNA not biological samples. We are not a clinical lab so we were just looking for residual RNA on doorknobs and things.

**Was this safe?**
 Yes. The students wore full personal protective equipment and disposed of samples as biological waste and handled no clinical or amplified samples. They never handled any virus. So, it was safer than a trip to the grocery store.

**What did they find?**

They found low levels of residual SARS-CoV2 RNA on campus at three sites.

**What does it mean?**

It indicates that probably someone with COVID passed through late summer or early fall. Only one sample was high enough to possibly have any virus present and that was borderline and probably not infectious. Most of the samples were negative.
